# Heroic Death: A Melancholic Existentialist Psychobiography of Jacques De Molay

**DOI:** 10.5964/ejop.17755

**Published:** 2025-11-28

**Authors:** Paul J. P. Fouché

**Affiliations:** 1 Department of Psychology, Faculty of the Humanities, University of the Free State, Bloemfontein, South Africa; University of Johannesburg, Johannesburg, South Africa

**Keywords:** Jacques de Molay, heroism, mortality, psychobiography, existentialism

## Abstract

This psychobiography aimed to reconstruct the life and death of Jacques de Molay (1243–1314), with particular focus upon his heroism as a mitigating psychological mechanism against the terror of death. Jacques de Molay was purposively sampled as subject. He was the Grand Master of the Knights Templar, an order of knighthood founded during the Crusades and dedicated to the mission of protecting Christian pilgrims and defending the Holy Land. He was confronted by betrayal; arrest; torture; confessions of heresy under duress; and public execution. Jacques de Molay’s life offers an exemplary illustration of the melancholic existentialist theory of Ernest Becker, who posited that mortality creates profound existential anxiety. This drives individuals to seek meaning in a ‘heroic struggle’, as an anxiety-buffering mechanism, to stifle the terror of death and dying. This psycho-historical analysis was conducted via the methodology of psychobiography. Publicly available historical and biographical data sources were utilized, and significant evidence across the lifespan of de Molay were extracted using the indicators of biographical salience promulgated by Irving Alexander. Salient themes and events were interpreted via Becker’s melancholic existential theory, with particular emphasis upon heroism. Jacques de Molay’s role as Grand Master represented his heroic dedication to the Knights Templar and their mission. The torture he endured and his refusal to betray the Templars’ ideals exemplified a heroic struggle against mortality. Instead of capitulating to terror and falsehood, de Molay faced his death maturely, thereby achieving existential authenticity and symbolic immortality.

*Heroism.…is a reflex to the terror of death*

- Ernest Becker (1973, p. 11).

Dying and death are experiences that evoke arcane psychological, religious and spiritual responses within humans. In psychology, the study of death and dying focuses on how individuals manage their mortality, express grief, and acquire a sense of meaning through loss. Religion provides comfort in beliefs of life after death, and in turn, spirituality offers worldviews of mortal transcendence (Tomer et al., 2008; Upenieks, 2023). From the viewpoint of transpersonal and existential psychology, the study of death and dying addresses the transformative potential of individuals’ everyday awareness of the role of death (Tomer et al., 2008; Wong, 2017). Research on death and dying has steadily increased within various scientific fields and undertaken from interdisciplinary approaches (e.g., Arrowood et al., 2023; Borgstrom & Ellis, 2017; Canzona et al., 2023; Cox & Thompson, 2021; Mayer, 2021; Meagher & Balk, 2013; Shackelford & Zeigler-Hill, 2019). The assumptions and beliefs of humans regarding death, impact not merely upon their experience of living, but also upon their transition through dying and death as a possible final developmental life-stage (Cox & Thompson, 2021; Kastenbaum, 2018).

Psychobiography, as a sub-field of psychohistory (Anderson, 2024; Anderson & Ponterotto, 2025; Ponterotto, 2025), is well-suited to provide for an in-depth historical analysis and psychological reconstruction of individuals’ lives across the lifespan (Citlak, 2023; Mayer & Kőváry, 2019). Psychobiographers primarily utilize psychological theories and apply these to the biographical and historical data on a significant individual’s life (Simonton, 1994). The aim is to explore, analyse and reconstruct various characteristics or attributes of extraordinariness across the lifespan of the subject. Psychobiography undertakes this against the background of the sociocultural and historical context of the subject (Fouché & van Niekerk, 2010; Mayer, Banai, & Stefanidis, 2025, Mayer, van Niekerk, & Banai, 2025). Recently, psychobiographers have illustrated the value in the use of interdisciplinary theoretical approaches and methodologies in undertaking psychobiography (Banai & Mayer, 2024; Martin, 2024; Mayer et al., 2023; Mayer & van Niekerk, 2024). Psychobiographies could provide for rich qualitative descriptions and contextual psychohistorical insights into how individuals deal with death and dying (Mayer, 2021; Mayer et al., 2021). Furthermore, the value of the practical applications of the lessons learned from psychobiographies of extraordinary individuals, to contemporary life, have previously been highlighted (Mayer & Fouché, 2021; Nel et al., 2024). This latter value coincides with the current chief-editor of Europe’s Journal of Psychology, Johannes Karl’s (2023) editorial, titled: ‘Preserving the flame: The past, present, and future of EJOP’. In this editorial the editor makes a call for a stronger focus upon contemporary practical applications, within publications, which support readers in the more applied implementation of lessons learned from authors’ research.

This psychobiography aimed to uncover, reconstruct and illustrate the life and death of Jacques de Molay (1243–1314), with particular focus upon heroism as a mitigating psychological mechanism against the terror of death, from the melancholic existentialist approach of Ernest Becker (1973, 1975). Particular attention was paid to the heroic role of de Molay in mitigating his death-anxiety and steadfastly face a heroic death. He remained loyal to his beliefs and the mission of the Templars even in the face of his own horrific death (Knight & Lomas, 1997). Jacques de Molay was the 23rd Grand Master of the Knights Templar, an Order of knighthood founded during the 12th century Crusades and dedicated to the mission of protecting Christian pilgrims and the Holy Land (Barber, 2012; Martin, 2004). He is viewed by many to be one of the most famous Templars in history (Gardner, 1996; Grishin, 2013). A brief historical sketch of his life and death is provided after the below discussion on Ernest Becker’s theory.

## Ernest Becker’s Melancholic Existentialist Theory

Ernest Becker (1924–1974) was born in Springfield, Massachusetts. After completing military service, he attended Syracuse University in New York. Thereafter, receiving his bachelor’s degree, he worked for the United States embassy in Paris. He returned to Syracuse University and pursued his post-graduate studies in anthropology and received his PhD in 1960. He spent most of his career in academia and found inspiration from interdisciplinary perspectives on humanity. He was influenced by the works of Alfred Adler, Carl Jung, Søren Kierkegaard, Charles Darwin, Sigmund Freud, Erich Fromm, and Otto Rank (Fisher, 2020; Munley & Johnson, 2003). Becker wrote nine books (Carveth, 2004), with his most notable works titled The birth and death of meaning (1971); The denial of death (1973), for which he posthumously received the 1974 Pulitzer Prize; and his final manuscript, Escape from evil (1975), that his wife Marie published for him after his death (Munley & Johnson, 2003).

As a melancholic existential anthropologist, Becker’s core argument entails that humans possess a formidable death-anxiety, and that heroism is a reflex in response to the terror of death. According to Becker, heroism can be extrapolated from a variety of cultural systems, that are either primitive, magical, secular, religious or scientific. The only requirement is that the cultural system should allow humans the opportunity to play a heroic-role that provides them with a sense of specialness, self-esteem, and meaning (Becker, 1973; Mayer et al., 2021). Becker posited that the fear of death is not continuously present in human mental awareness, since if it were, individuals would be incapacitated to function ‘normally’. He postulated that culture, worldviews, and heroism aim at providing humans with a sense of value and hope of transcending death (Becker, 1973; Evans, 1992; Vail et al., 2012; Vail & Routledge, 2020). According to Becker (1968, 1969, 1971, 1973) humans employ psychological mechanisms to navigate their struggle against mortality. These include the following:

- Cultural mechanisms of death-denial such as political power, a strong national identity, artistic achievements and established social norms.
- Religious beliefs, with a promise of an afterlife or spiritual continuation.
- Technological advancements in medicine, artificial intelligence, and biotechnology that offer the promise of extending human life or achieving digital immortality (Hollanek & Nowaczyk-Basińska, 2024).
- A sense of heroism through accomplishments, which boosts self-esteem and transcends mortal limitations.

Becker died from colon cancer during 1974 in British Columbia, where he held a professorship at Simon Fraser University (SFU), (Kramer, 2007; Martin, 1997; Martin, 2014). After Becker’s death, The Ernest Becker Foundation was founded (Ernest Becker Foundation, n.d.). The foundation focused on multidisciplinary research, particularly at the interface of the humanities and religion (Hayes et al., 2010; Martin, 1997). A captivating and illuminating essay on Ernest Becker during his last years (1969–1974) of academic scholarship and his dying from cancer, was produced by Professor Jack Martin (2014), from SFU, and published in the Journal of Humanistic Psychology. Becker’s years at SFU are revealed as a professional career, personal existential struggle, and a period that was both heroic and tragic (Martin, 2014). During this period Becker’s academic work ironically merged with his impending death, as both came to a finality (Kramer, 2007; Martin, 2014).

Becker’s work had inspired the development of Terror Management Theory (TMT), under the auspices of renowned Sheldon Solomon, Jeff Greenberg and Tom Pyszczynski (Liechty, 2002). Other related research includes the mortality salience hypothesis (Arrowood et al., 2023; Greenberg et al., 2004; Juhl, 2019; Koole et al., 2006; Rosenblatt et al.,1989), according to which death-anxiety drives individuals to adopt worldviews that endorse their worthiness and buffer their awareness of mortality (Mayer et al., 2021; Pyszczynski et al., 2010, 2015). According to Carveth (2004), the documentary film based on Becker’s work, Flight from death: The quest for immortality (Shen, 2005), also contributed to much interest in melancholic existentialism after its release.

## A Brief Historical Sketch of the Life and Death of de Molay

### The Early Life of de Molay

Jacques de Molay was born around 1243 in Molay, Haute-Saône, in the County of Burgundy. The exact year of his birth is a matter of debate. According to some sources, he was 21 years old when he was knighted in 1265 and was about 70 years old when he was executed in 1314 (Knight & Lomas, 2001; Singh, 2024). This implies his birth year to be 1243 or 1244. Credible scholars indicate his year of birth as 1243 (Demurger, 2002, 2009; Frale, 2009; Martin, 2004; Singh, 2024). Little is known about his family and early years, but he was likely born into a family of minor nobility (Barber, 2012; Haag, 2008). Jacques de Molay joined the Knights Templar during 1265 and was initiated as a member of the Knights Templar in a chapel at the Beaune House, by Humbert de Pairaud, the dignitary of the Knights Templar in France and England. The ceremony was attended by Amaury de la Roche, who served as the Templar Master of the province of the France (Haag, 2008; Knight & Lomas, 2001). As the ceremonial ruling required, the novice initiate was received in front of all the members of the Order who were present, his duties and vows were read to him, and then the appropriate Order mantle was bestowed upon him (Gardner, 1996; Upton-Ward, 1992). The Templars were allowed to wear a white mantle with a Templar blood cross on it. They were obliged to grow their beards to separate them from other fraternities (Gardner, 1996). Around 1270, de Molay travelled to the Middle East (i.e., Crusader states), known as Outremer. Little is known about his activities in the ensuing 20 years. Literature indicates that he quickly rose through the ranks due to his dedication to the Order’s mission, beliefs and his loyalty to the papacy and European royals (Martin, 2004; Musarra et al., 2022).

### The Grand Master of the Knights Templar

In 1291, the Crusaders lost the city of Acre, to the Egyptian Mamluks. Acre, an ancient city also known as Akko, was the principal port and stronghold during the late Crusader period. It was located on the Mediterranean coast in the northern part of today’s Israel (France, 2018). The most enduring Mamluk grouping was the knightly military class in medieval Egypt, which developed from the ranks of slave-soldiers (Musara et al., 2022; Singh, 2024). After Acre was captured, the Catholic Europeans (i.e., the Franks) retreated to the island of Cyprus. It served as their new base due to their declining power within the Middle East. Cyprus now served as the departure point for military excursions by the Crusaders against the Egyptian Mamluks. The Christian West was unable to fully regain the Holy Land, and the Muslim East required more planning and resources to conquer the kingdoms of Armenia and Cyprus (Singh, 2024). During this period, the Templars were still led by Thibaud Gaudin, their 22^nd^ Grand Master. In 1291, during a meeting in Cyprus, de Molay revealed the reforms he planned to bring to the Order if he were to replace Gaudin as the Grand Master. His initial aim was to fortify the defense of Cyprus and reorganize and strengthen the Templar forces. During 1292, Gaudin passed away, and in the absence of any other strong contenders, de Molay was elected as the 23^rd^ Grand Master (Knight & Lomas, 1997; Singh, 2024; Upton-Ward, 1992).

Jacques de Molay had to reassure his Knights Templar of their significance and reignite enthusiasm amongst his Order after the loss of Acre (Singh, 2024). He decided to undertake resolute diplomatic action (Josserand, 2020). He and his Templars undertook two journeys to the West. The first journey took place in the winter of 1292–1293, and a second one, from Spring 1293 to Autumn 1296. For approximately three and a half years, de Molay became more acquainted and diplomatically linked with powerful leaders such as King Charles II of Naples and Pope Boniface VIII, but also Edward I of England, and James II of Aragon. By visiting these European kingdoms and the papacy, de Molay hoped to harness more political and military support for the Templars (Haag, 2008; Josserand, 2020; Nicholson, 2001). He was successful in acquiring the permission from some of the rulers for the transport of supplies to Cyprus (Gardner, 1996; Singh, 2024).

Between 1299 and 1303, de Molay orchestrated a new attack on the Mamluks. The plan was a collaboration between the Christian military Orders, the King and nobility of Cyprus, the forces of Cilician Armenia, and a new possible ally, the Mongols. The coalition’s mission was to reclaim the coastal city of Tortosa in Syria (Martin, 2004; Musarra et al., 2022). In 1300, de Molay and the Templars took part in excursions along the Egyptian and Syrian coasts. The Cypriots also amalgamated a larger force to mount an attack on Tortosa. They used the island of Ruad, close to the city of Tortosa, to bridge their attacks on the mainland. However, the Crusaders, which included the military Orders of the Knights Templars, Hospitallers and their allies, suffered a surprisingly momentous defeat at the Siege of Ruad in September 1302. They lost the island which served as their crucial last bridgehead close to the mainland (Haag, 2008, 2012; Singh, 2024). This defeat diminished the Crusaders’ ability to protect the Holy Land (Barber, 2012; Martin, 2004).

### Dispute With King Philip IV of France

In 1305, Pope Clement V, who had newly assumed the papal office, enquired as to the viewpoints of the leaders of the military Orders on a planned new Crusade, as well as the unification of their Orders. Jacques de Molay submitted memoranda on both issues (Gardner, 1996). While he supported the notion of a new Crusade, he refused the suggestion of merging the Orders between the Knights Templar with the Knights Hospitaller (i.e., an Order that cared for injured pilgrims). The Grand Master of the Hospitallers, Fulk de Villaret, likewise resisted the proposal of amalgamation. Both these Orders had their own unique identity and mission that the two Grand Masters did not want to see attenuated by a merger (Knight & Lomas, 1997; Singh, 2024). However, Philippe had accumulated an enormous debt to the Templars. According to Gardner (1996), he was practically bankrupt and feared the Templars’ political and esoteric might. Philippe advocated for the Orders to be merged under his leadership, which would have made him Rex Bellator or the War King. Jacques de Molay vehemently vetoed this proposal (Demurger, 2009; Gardner, 1996).

To complicate matters, Philippe was disputing the papacy at the time. His rupture with Pope Boniface VIII was due to a power-struggle between them. Phillipe then briefly imprisoned Pope Boniface VIII, whereafter he died soon after his release. There are also claims that Philippe had the papal successor, Pope Benedict XI, poisoned. However, Pope Benedict’s successor, Pope Clement V was French, and Philippe commanded considerable political power over Pope Clement V (Gardner, 1996). It was now that accusations were made against the Knights Templar of deeds of misconduct during the initiation ceremony of new members. These accusations were mainly forthcoming from expelled former members of the order (Gardner, 1996; Knight & Lomas, 1997). Jacques de Molay requested Pope Clement V to conduct a formal investigation into the matter, hoping that the Order would be quickly exonerated of the charges of misconduct (Knight & Lomas, 2001; Singh, 2024).

### Arrest, Torture, Confession and Execution of de Molay

Initially, the Templars were faced with only a few charges including the renunciation of, and spitting on, the cross during the Order’s initiation ceremonies. The number of charges increased amidst all the allegations that were spread. Philippe ordered his lawyers, soldiers and henchmen to arrange for the arrest of all Templars in his kingdom. His agents executed the order at dawn on 13 October 1307 (Gardner, 1996; Haag, 2012). Many Templars, including de Molay, were apprehended and charges of heresy were levelled against them (Barber, 2006; Demurger, 2002, 2009; Grishin, 2013). After investigations were conducted under the decree of Pope Clement V, all the arrested Templars in France, including de Molay, were interrogated by Philippe’s agents, who wanted to crush the Order’s power and steal the wealth they had amassed from their battles during the Crusades. Jacques de Molay was tortured by Philippe’s agents in late October and was forced, under duress, to admit to additional charges of heresy. Wiliam Imbert, the Chief Inquisitor of France, was “deeply versed in all inquisitorial arts and practices” (Knight & Lomas, 1997, p. 166). He was ordered by Philippe with the task of extracting an immediate confession from de Molay, by whatever means he considered effective (Knight & Lomas, 1997). The additional charges were claimed to involve the denial of Christ and abusing the crucifix in sacrilegious initiation rituals (Knight & Lomas, 2001). Paid witnesses were called to give evidence against the Order, and some bizarre statements were obtained, which included practices such as necromancy, abortion, blasphemy, and the black arts (Gardner, 1996). On 12 March 1312, a papal decree forcibly suppressed the Order of the Knights Templar (Gardner, 1996). Knowing that Pope Clement V was a pawn of Philippe, and that both parties would want a share in the Templar Order’s wealth and treasures, de Molay secretly arranged for many Templar treasures, which were hidden in vaults in Paris, to be removed in a fleet of 18 galleys, from La Rochelle. Philippe was unaware of this, and most of these treasure ships and accompanying Templars sailed to Scotland where they found sanctuary under King Robert the Bruce, and the Scottish nation (Gardner, 1996; Haag, 2008, 2012).

Of interest to note, is that those responsible for de Molay’s torture, were under strict orders not to kill the Grand Master (Gardner, 1996). According to Knight and Lomas (1997, 2001), de Molay had to experience a similar torture to that of Jesus Christ’s crucifixion. A detailed graphic description of de Molay’s interrogation and torture in the Paris Temple is provided by Knight and Lomas (1997, pp. 165–169). According to Singh (2024) the Pope still sought to interrogate de Molay himself. Jacques de Molay subsequently retracted his confession in front of papal agents. However, in another interrogation, conducted in the presence of both papal and Philippe’s agents, he reverted to his forced admissions of 1307. According to Church law, a relapsed heretic had no prospect of a second opportunity. The punishment for relapsing was that of being burnt to death (Gardner, 1996). Jacques de Molay and his loyal fellow Templar, Geoffroi de Charney, were publicly executed at the stake on a scaffold near the Notre Dame cathedral on 18 March 1314 (Josserand, 2021; Martin, 2004). Jacques de Molay met his death with an apparent calm and serene demeanor (Knight & Lomas, 2001; Singh, 2024). Rumours exist that de Molay, prior to dying, summoned Philippe and Pope Clement V to meet God within a year, where they would be judged for their crimes (Gardner, 1996; Knight & Lomas, 1997). Both Philippe and the Pope died within a year of de Molay’s execution (Gardner, 1996).

## Research Design and Methodology

Psychobiography can be described as a biography written on a significant individual, or individuals, utilizing mostly a psychological or well-suited and relevant theory (Mayer, Banai, & Stefanidis, 2025, Mayer, van Niekerk, & Banai, 2025; Ponterotto, 2017a, 2017b). It represents a longitudinal case-study of an extraordinary individual, over the lifespan (Fouché, 2015). Psychobiography is a subdivision of psychohistory (Ponterotto, 2015), with the psychobiographer utilizing psychological theory and psychohistorical analysis to explore and reconstruct the life of the subject (Nel et al., 2024) within the sociocultural context in which they lived (Fouché, 2025; Mayer & van Niekerk, 2024).

### The Psychobiographical Subject: Jacques de Molay

Jacques de Molay was chosen as the subject, utilising a non-probability, purposive sampling technique. Purposive sampling relies on the researcher's judgement in determining the desired characteristics or attributes to be investigated and to ensure the richness of data (Creswell, 2013). The life and death of de Molay provide for many lessons to be learned for contemporary studies on death and dying, and also for humanity’s contemporary existential Angst and fear of geopolitical warfare and the inevitable suffering, dying and death. These lessons learned are discussed further in the “Conclusion, Recommendations and Practical Application” section of this article. According to Frale (2009) and Singh (2024), the significance of de Molay could be ascribed to the following historical points, namely:

- de Molay was the 23^rd^ and last Grand Master of the Knights Templar.
- He was renowned for his commitment to the Templar Order and its mission, and later in life, also his diplomacy (Josserand, 2020).
- Despite facing persecution and being burned at the stake, de Molay remained loyal to his beliefs and the ideals of the Knights Templar.
- de Molay’s courage in the face of death has inspired countless creative works of literature, art, and film, that also inspire contemporary audiences of the day.

In addition, Laurence Gardner (1996), author of Bloodline of the Holy Grail, highlighted the following significance that the Knights Templar and de Molay held in history, namely:

- Knowledge of the placement of the hidden Ark of the Covenant.
- The Templars’ knowledge of the secret geometry of King Solomon’s masons, especially that of the architect, Hiram Abiff, the chief designer of the Temple of Jehovah.
- Their skilled entrepreneurship as bankers, taking deposits from pilgrims, knights and nobles who could then withdraw their money at any Templar house from London to Jerusalem. This meant that the wealthy could avoid the risk of robbery during their travels.

### Data-Source Collection

An initial literature search, using EBSCOHost, revealed that publicly accessible data-sources on de Molay existed, except for the sparse information on his early and personal life. Only publicly available data sources were utilised for data collection, extraction, and analysis. These included credible academic articles, books and biographical, historical and digital online sources. The majority of available data sources were centered around the following: Firstly, de Molay’s dedication to the Knights Templar and the Crusades. Secondly, his planned reforms to the Order in the waning days of the Crusades. Thirdly, the growing reluctance of European royalty to support the Crusades. Fourthly, de Molay’s dispute with King Philip IV (i.e., Philippe) who insisted the Knights Templar merge with the Knights Hospitallers, under Philippe’s command (Demurger, 2009). Lastly, his arrest, inquisition, torture and execution, which appears to have received most historic attention (Barber, 2006, 2012; Frale, 2004; Grishin, 2013; Josserand, 2021; Knight & Lomas, 1997).

Although the quantity of available sources is deemed of importance, the rigor and trustworthiness of the data-sources are of more value to enhance methodological integrity (Levitt et al., 2017; Mayer & Kőváry, 2019). Credible historians and researchers were identified who were well-versed in Templar history and the life of de Molay. Some of these notable researchers include: (a) Alain Demurger (2002, 2009); (b) Malcolm Barber (2006, 2012); (c) Helen Nicholson (2001); (d) Philippe Josserand (2020, 2021); and (e) Laurence Gardner (1996), the chivalric genealogist and Royal Jacobite Historiographer. Of special interest is Barbara Frale (2004, 2009), an Italian paleographer at the Vatican Secret Archives. A publication of her work appeared in the Journal of Medieval History in 2004, titled, ‘The Chinon chart: Papal absolution to the last Templar, Master Jacques de Molay’. Frale documented that the discovery of the papal records of the hearings of the Templars and de Molay, contain proof of de Molay’s papal absolution by Pope Clement V. Notwithstanding, Philippe enforced the execution of de Molay. Frale’s interest in the history of the Shroud of Turin is also of relevance to this study, due to its possible connection to the post-torture, bodily image left on the shroud. Knight and Lomas (1997, 2001) have speculated that the Shroud of Turin could be the linen cloth used to enshroud, the still alive de Molay’s body, after his medieval torture, (which was a parody of the crucifixion of Jesus Christ). However, much controversy remains regarding the true image formation on the Shroud of Turin (e.g., Allen et al., 1999).

### Data Extraction and Analysis

A psychobiography has rigor and methodological integrity, if the researcher can identify and extract relevant, and salient evidence on the subject and apply it to the psychological theory utilised in the study, and vice versa. Generalising the case findings to the theory, utilized to study the subject, is referred to as analytical generalisation (Yin, 2018). The first strategy used by this researcher entailed the systematic extraction of salient thematic evidence for further theoretical interpretation. Alexander’s (1988, 1990) biographical indicators of thematic saliency (i.e., uniqueness, negation, emphasis, primacy, frequency, error or distortion, isolation, incompletion, and omission) were utilised for the identification and extraction of salient themes or units for analysis. As a second strategy, the researcher questioned the extracted themes or evidence for its relevance to the study’s aim. The researcher undertook this by asking the following question: Which sections of the collected data-sources and extracted salient themes of evidence, will allow for an in-depth understanding, reconstruction and illustration of de Molay’s heroic death, especially his psychological mechanism of heroism, used to mitigate his death-anxiety, from the melancholic existentialist viewpoint of Becker?

### Methodological Integrity, Rigor and Trustworthiness

Psychobiographies are no longer critiqued for their once contested methodological integrity, and are increasingly incorporating anthropological, sociological, spiritual and historical theories and methodologies into their undertaking (Citlak, 2023; Mayer, 2023). To ensure psychobiographical rigour and trustworthiness, four quality criteria should be considered, namely: confirmability, credibility, transferability, and dependability (Fouché & Strauss, 2024; Yin, 2018). Confirmability was ensured by using the constructs and propositions proposed by Becker’s (1969, 1973, 1975) theory to explore the psychological mechanisms that humans employ to deny and mitigate the terror of death. These constructs and propositions included: (a) cultural mechanisms of denial; (b) religious beliefs; (c) heroism and self-esteem; and (d) death-transcendence (Becker, 1969, 1973, 1975). Credibility was ensured through data triangulation (Yin, 2018). A range of publicly available data sources were included and analysed according to the inter-connected constructs and propositions posited within Becker’s theory. Transferability in psychobiography is ensured when the study’s findings can be analytically generalised by comparing the psychobiographical findings to an established theory that was utilized in the case-analysis (Fouché, 2025). Significant findings on de Molay’s dying and death were compared to the melancholic existential theory of Becker. Dependability, also referred to as auditability, is ensured when a study can be duplicated and produce similar results. Dependability was strengthened through the systematic and consistent implementation of data collection, extraction and thematic analysis procedures. The overall methodological integrity was maintained by following the recommendations of Levitt et al. (2017).

### Ethical Considerations

Valuable advice regarding ethical considerations in psychobiography have been provided by Ponterotto and Reynolds (2019) and Ponterotto (2025). Only publicly available data sources were used to ensure the privacy rights of possible relatives of de Molay and significant others referred to in the psychobiography. No credible evidence could be identified from the data sources to indicate that de Molay had children of his own.

Furthermore, an online genealogical website (Geni, 2025) indicates there are no Y-DNA results for relatives in the paternal line of de Molay, and no mitochondrial DNA results for the maternal line. Autosomal DNA results also indicate no known existence of close relatives of de Molay. This psychobiography adhered to the recommended best practices in psychobiography proposed by Ponterotto (2014), as well as the implementation of legal and ethical guidelines that psychobiography should adhere to (Ponterotto & Reynolds, 2019). Ethical clearance for this study was obtained from the author’s institutional review board.

## Findings and Discussion

Jacques de Molay is shrouded in history as a famous Knights Templar who steadfastly and heroically faced his horrific death (Gardner, 1996; Knight & Lomas, 1997). His mission was to protect Christian pilgrims and the Holy Land. The Templars undertook Crusades, during which the Knights and de Molay were inevitably confronted with frequent scenes of dying and death (Gardner, 1996). The author of this article speculates that the Crusades and military excursions most probably could have afflicted the Templars with psychological trauma. However, this could not be credibly verified from the sources of data. Jacques de Molay had for most of his adult life experienced exposure to violence and death, justified by his loyalty to the papacy and the mission, ideals and values of the Knights Templar (Grishin, 2013). Becker (1973) referred to this as the human need to play a heroic role and be of value to the cultural system they serve. Each culture offers unique customs and beliefs that provide guidelines, that serve as framework for justified heroism (Becker, 1975; Munley & Johnson, 2003). It may be that de Molay, in his heroic role as Templar, and eventually that of Grand Master in loyal service of the papacy, provided him with a sense of heroic self-esteem to justify his duties and loyally execute his role as Crusader Knight. This may also have mitigated the terror of his own impending death, albeit ironically at the hands of those he loyally served. On 13 October 1307, de Molay and other accused Templars were arrested and subjected to interrogation and torture to forcefully elicit confessions to heresy and crimes against Church dogma (Gardner, 1996; Knight & Lomas, 2001).

Becker's theory provided for an ideal approach to explore, reconstruct and illustrate de Molay's life and heroic death. His life and death are emblematic of the existential struggle endured by individuals confronting injustice and existential despair in contemporary society. The dying and death of de Molay, seemed to embody three clear melancholic existentialist themes, namely:

- *Alienation:* de Molay's betrayal and imprisonment reflect the existentialist notion of alienation from the culturally expected norms of the papacy, church and the European royals he served (Demurger, 2002; Frale, 2009).
- *Authenticity:* his final refusal to accept false accusations of heresy and his ultimate recantation of confessions he made under duress (Knight & Lomas, 1997; Nicholson, 2001), demonstrated his commitment to truth and authenticity, which involves committing to one’s true inner values and beliefs, rather than merely conforming to expected societal norms (Zimmerman, 1983)
- His *Sense of Despair* and *Search for Meaning*: de Molay's suffering and death highlight the existential struggle to find meaning in the face of injustice, during times of non-sensical war, power-politics, religious self-righteousness, and the greed for wealth (Gardner, 1996; Singh, 2024).

Jacques de Molay’s leadership coincided with a period of much challenge for the Templars, as European support for the Crusades waned and political tensions increased (Gardner, 1996; Knight & Lomas, 2001). His existential struggle began in earnest when Philippe, deeply in debt to the Templars, sought to disband the Order and seize its wealth (Barber, 2006; Gardner, 1996). He endured medieval torture, and as a result, he initially confessed to heresy, but later recanted, maintaining his and the Order’s innocence. This act of defiance highlighted his commitment to the Templar beliefs, ideals and values. Jacques de Molay’s trial was a parody, marked by political machinations and a predetermined outcome (Gardner, 1996; Singh, 2024). On 18 March 1314, de Molay was burned at the stake in Paris as a public display, intended to serve as a warning to others. His final moments were marked by dignity and courage (Knight & Lomas, 1997; Singh, 2024). However, rumors have it that he imprecated Philippe and Pope Clement V, both of whom died within a year of his execution (Gardner, 1996; Haag, 2008, 2012). His death, ordered by Philippe (Gardner, 1996), underscores the existentialist notion of absurdity and injustice represented by the conflict between the human desire for meaning and an indifferent universe (Munley & Johnson, 2003). Molay’s execution highlights the existentialist view that individuals must navigate a world where rationality and fairness are not guaranteed, and where one's existence can be abruptly ended without reason or justification (Becker, 1973, 1975). His death is a poignant example of existential despair and humanity’s confrontation with death and nihilism (Becker, 1973). The findings illustrate the role of heroism as mitigating psychological mechanism in dealing with death-anxiety through creating a sense of purpose and meaning that transcends finite existence (Becker, 1961, 1962, 1964, 1967, 1975; Holloway, 2017; Kenel, 1988; Schimel et al., 2007; Snyder & Bowers, 2015).

### Conclusion, Recommendations and Practical Applications

The legacy of de Molay continues to inspire and evoke reflection on the nature of suffering, death and dying. His life and embracement of death, offers historical lessons to be applied to contemporary society and human dying and death. It provides suggestions for plausible further refinement of Becker’s theory of melancholic existentialism. Furthermore, it offers practical ways to apply lessons learned from de Molay’s heroic death to humanity in contemporary society. His courage in the face of his betrayal, the agonizing torture he endured and his horrific death at the stake, are indicative of a blend of emotions, which calls for the refinement of Becker’s melancholic existentialist theory. A first plausible and recommended refinement could be that of a serene-melancholic existentialist framework, within which death and dying could be viewed as a serene practice, that involves a blended emotionality (i.e., the acceptance of mortality within a serene melancholy of existential authenticity). This ability to experience serenity, in conjunction with melancholy, that results from the recognition of life’s impermanence, and biological decay, constitutes a blended emotion. The latter is closely embodiment in the Japanese worldview called Wabi Sabi (Juniper, 2003; Koren, 1994). Wabi Sabi (i.e., the simple beauty of serene melancholy) could offer a practical modus operandi for understanding and integrating the seemingly dichotomous forces of living and dying concurrently. From this perspective, death-anxiety is not pathological, but a natural response to the realization of life’s impermanence (Juniper, 2003). Contemporary existentialists claim that the acceptance of life’s impermanence, provides for a pathway to a more authentic living and death (Transpersonal Psychology, n.d.).

The death of de Molay solidified his legacy and remembrance (Knight & Lomas, 1997), thus achieving a form of symbolic immortality (Becker, 1973; Holloway, 2017). The importance of giving more attention to the notion of legacy is a second recommendation this author calls for in refining melancholic existentialist theory. This could be represented by a melancholic existentialist approach that more definitively incorporates the notions of legacy and remembrance. The concept of legacy encompasses the ways in which individuals aspire to be remembered after their death, including their values, achievements, and the impact they had upon society. Legacy and remembrance play a crucial role in shaping one’s identity and purpose, as individuals reflect on what they want to leave behind for posterity (Snyder & Bowers, 2015). Legacy serves as a vital psychological mechanism that influences how individuals approach their mortality and experience their dying and death (Holloway, 2017). Leaving a meaningful legacy can provide a sense of purpose, particularly in the later stages of the human lifespan, or when confronted with dying and death. By contemplating their legacy, individuals engage in a process of self-reflection that facilitates a greater acceptance of their mortality (Snyder & Bowers, 2015). The building of legacy could be practically implemented in daily life by regular self-reflection upon what achieved value we have added to others and our own lives. Furthermore, by reflecting upon what remembrance and value can still be created, not just for ourselves, but for others through our altruistic deeds of heroism and an eventual heroic death.
